# Barriers to identifying eating disorders in pregnancy and in the postnatal period: a qualitative approach

**DOI:** 10.1186/s12884-018-1745-x

**Published:** 2018-05-15

**Authors:** Amanda Bye, Jill Shawe, Debra Bick, Abigail Easter, Megan Kash-Macdonald, Nadia Micali

**Affiliations:** 1 Population, Policy and Practice, UCL Great Ormond Street Institute of Child Health, 30 Guilford Street, WC1N 1EH, London, UK; 2 Centre for Implementation Science, Health Service and Population Research, Institute of Psychiatry, Psychology & Neuroscience, King’s College London, London, UK; 3 Department of Midwifery, Florence Nightingale Faculty of Nursing, Midwifery & Palliative Care, King’s College London, London, UK; 4 Institute of Health and Community, University of Plymouth, Devon, UK; 5 Child and Adolescent Psychiatry Division, Department of Child and Adolescent Health, Geneva University Hospital, Geneva, Switzerland; 6 Department of Psychiatry, Faculty of Medicine, University of Geneva, Geneva, Switzerland; 7 Department of Women and Children’s Health, School of Life Course Sciences, Faculty of Life Sciences and Medicine, King’s College London, London, UK

**Keywords:** Eating Disorders, Pregnancy, Barriers, Disclosure, Identification, Qualitative research

## Abstract

**Background:**

Eating Disorders (ED) are mental health disorders that typically effect women of childbearing age and are associated with adverse maternal and infant outcomes. UK healthcare guidance recommends routine enquiry for current and past mental illness in antenatal and postnatal care for all women, and that pregnant women with a known ED are offered enhanced monitoring and support. Midwives and health visitors are ideally placed to identify and support women with ED as they are often the primary point of contact during the antenatal and postnatal periods. However, research on the barriers to identifying ED in the perinatal period is limited. This study aimed to understand the barriers to disclosure and identification of ED in pregnancy and postnatally as perceived by women with past or current ED, and midwives and health visitors working in the UK National Health Service.

**Methods:**

Two studies were undertaken: mixed-measures survey of pregnant and postnatal women with current or past ED; focus groups with student and qualified midwives and health visitors.

**Results:**

Five themes emerged on the barriers to disclosure in pregnancy as perceived by women: stigma, lack of opportunity, preference for self-management, current ED symptomatology and illness awareness. Four themes were identified on the barriers to identification of ED in pregnancy and in the postnatal period as perceived by health professionals: system constraints, recognition of role, personal attitudes, and stigma and taboo.

**Conclusions:**

Several barriers to the identification of ED during and after pregnancy were described, the main factors were stigma and poor professional training. Perinatal mental health is becoming increasingly prioritised within national policy initiatives; however, ED continue to be neglected and increased awareness is needed. Similarly, clinical guidance aimed at responding to the rising prevalence of obesity focus on changing nutrition but not on assessing for the presence of ED behaviours that might be affecting nutrition. Improving education and training for health professionals may contribute to reducing stigma and increase confidence in identifying ED. The barriers identified in this research need to be addressed if recognition and response to women with ED during the perinatal period is to improve.

**Electronic supplementary material:**

The online version of this article (10.1186/s12884-018-1745-x) contains supplementary material, which is available to authorized users.

## Background

Eating Disorders (ED) are a group of mental health disorders characterised by severe disturbances in eating behaviour that significantly impair health and psychosocial functioning, including Anorexia Nervosa (AN), Bulimia Nervosa (BN) and Binge-Eating Disorder (BED) [1]. ED typically affect women of reproductive age [2] and may affect between 5.1-7.5% of women during pregnancy, if subthreshold disorders are included [3–5]. Women with ED tend to experience a decrease in ED symptoms during pregnancy [6–9]. However, there is evidence that symptoms persist [7, 8] and of postnatal relapse [6, 8, 9]. Furthermore, depression and anxiety symptoms are common during pregnancy and postnatally for women with current and past ED [10, 11].

ED have been associated with various adverse pregnancy outcomes, for instance women with AN have higher risk of infertility, unplanned pregnancies, miscarriage, prematurity and low birth weight babies while women with BED have increased risk of higher birth weight babies [12–17]. There is growing evidence of the postnatal impact of maternal ED, such as difficulties with infant feeding [18–20], and behavioural and emotional problems in the infant [21–23].

Given the evidence of adverse outcomes for women and their infants, early identification of ED and appropriate antenatal and postnatal care are highly important. In the UK, National Health Service (NHS) maternity care is informed by a suite of guidance from the National Institute for Health and Care Excellence (NICE). NICE antenatal and postnatal mental health guidance [24] recommends routine enquiry about current and past severe mental illness with all women, and women with ED should be offered enhanced support and monitoring, and referred to specialist care if needed. Midwives and health visitors are ideally placed to identify and support women with ED as they are the primary point of contact delivering routine care for all women from pregnancy until the child is aged five. In a universal healthcare system such as the NHS where these regular routine appointments are provided, guidelines to support effective identification and management of women with ED should be implemented to reduce risk of poor pregnancy and birth outcomes, however evidence on uptake and use of guidance is limited.

To our knowledge, no previous studies have specifically investigated the barriers to identifying ED in the perinatal period. Evidence suggests that women with ED are often reluctant to disclose their ED to a health professional [25] and are unlikely to seek treatment [26], which may be due to feelings of stigma and shame [27, 28]. One UK-based qualitative study investigating women’s views of antenatal care found that women were reluctant to disclose their ED because they felt health professionals lacked ED knowledge and sensitivity in dealing with the disorder [29]. A few studies in the US have found poor routine enquiry and knowledge about ED behaviours and symptoms among clinicians, including obstetricians [30–32].

Subsequently, women with ED may go undetected during pregnancy and postnatally, with potential implications for adverse maternal and infant health outcomes if disorders are not managed appropriately. An in-depth understanding of barriers to the identification of ED during and after pregnancy which reflects the perspectives of women and health professionals is needed to assess and inform practice, including implementation of relevant guidelines in to practice.

### Objectives

To understand the barriers to disclosure and identification of ED in pregnancy and postnatally as perceived by women with past or current ED, and midwives and health visitors working in the UK NHS.

## Methods

Two studies were undertaken:

### Study 1

#### Design and setting

A mixed-measures survey of pregnant and postnatal women with current or past ED was conducted over a seven-month period. Women were recruited via a national parenting website, Netmums, which is a UK-based online parenting organisation with over 1.7 million members. Ethical approval was granted by the University College London’s Research Ethics Committee (Ref. 3735/002).

#### Recruitment and procedure

The study employed convenience sampling by inviting women to voluntarily take part in an online survey via a study advertisement on the organisation website. Before commencing the survey, women were asked to read the information about the study displayed on the website. To be eligible, women had to answer yes to “Do you have, or have you had an eating disorder?”, and respond to “How many children do you have?” with either the number of children or that they are currently pregnant. For women who were eligible and willing to take part, consent to participate was implied by virtue of survey completion. The survey was developed specifically for this study, and questions of interest were a combination of seven Likert-type scale questions and an open-ended question (see Additional file 1).

#### Participants

A total of 101 women completed the mixed-measures survey, the majority of whom were not currently pregnant and already had children and had experienced an ED in the past or currently (*n* = 92; see Table 1). The sample consisted of women from across the UK, with a range of age and educational attainment reflected.

**Table 1:** Study 1: Sample characteristics

Characteristics N (%)	*N* = 101
Age	
≤ 24	21 (21%)
25-35	55 (55%)
≧36	24 (24%)
*Missing*	*1*
Parity	
Currently pregnant	9 (9%)
1 child	39 (39%)
2 children	37 (37%)
≧3 children	16 (16%)
*Missing*	*–*
Location	
England	
London	16 (16%)
Midlands and East of England	18 (18%)
North England	26 (26%)
South England	24 (24%)
Wales	6 (6%)
Scotland	5 (5%)
Northern Ireland	3 (3%)
*Missing*	*3*
Education	
GCSE or equivalent	27 (27%)
A level or equivalent	28 (28%)
Degree or above	44 (44%)
*Missing*	*2*
Type of eating disorder	
Anorexia Nervosa	34 (34%)
Bulimia Nervosa	16 (16%)
Binge Eating Disorder	24 (24%)
Eating Disorder Not Otherwise Specified	25 (25%)
*Missing*	*2*
Any eating disorder symptoms experienced during pregnancy	
No	36 (36%)
Yes	64 (63%)
*Missing*	*1*
Eating disorder symptoms experienced during pregnancy	
Purging	12 (12%)
Binge eating	29 (29%)
Calorie restriction	31 (31%)
Excessive exercise	14 (14%)
Low weight	17 (17%)
Eating disorder symptoms that improved during pregnancy	
Purging	16 (16%)
Binge eating	21 (21%)
Calorie restriction	27 (27%)
Excessive exercise	12 (12%)
Low weight	15 (15%)
Health professional aware of eating disorder	
Yes	22 (22%)
No	62 (61%)
Unsure	16 (16%)
*Missing*	*1*
Informed health professional about eating disorder	
Yes	26 (26%)
No	72 (71%)
*Missing*	*3*

#### Analysis

Data were analysed using the thematic analysis approach described by Braun and Clark [33]. This approach is an inductive and iterative process involving six phases of analysis: familiarisation with data, generation of initial codes, searching for themes among codes, reviewing themes, defining and naming themes, and producing the final report. Data were independently coded by two researchers (AB and KT), both of whom were trained in qualitative research methods and analysis. The rating-pair familiarised themselves with the data and independently coded the complete data set to ensure reliability and thoroughness, ensuring full consideration could be given to patterns within the data. The pair discussed their codes together, with an 89% agreement being achieved by the rating-pair. At this stage, codes with similar information were grouped, discrepancies discussed, and agreement sought before amendments were made and emerging themes sought. The process was iterative throughout, with continual reference to the original data to validate and refine emerging themes. The themes were then clustered into subordinate themes, and finally superordinate themes. The research team contributed to the refining, naming and interpretation of the themes. Quotes that were illustrative of the themes and subthemes are presented in the results and additional files.

### Study 2

#### Design and setting

Focus groups with student and qualified midwives and health visitors were conducted over a seven-month period at participating universities and NHS hospital and community services in the South of England. Ethical approval was granted by the University College London’s Research Ethics Committee (Ref. 3735/001) and the Joint Research and Development Office for Great Ormond Street Hospital for Children NHS Foundation Trust & The UCL Institute of Child Health (Ref. 11BS33).

### Recruitment and procedure

A convenience sampling strategy was employed to recruit participants to the focus groups. Student midwives were recruited from across three universities, and student health visitors from one university. Qualified midwives were recruited from two hospital and community services, and qualified health visitors from one community service. Five focus groups were conducted, with one group per professional group, except for qualified midwives who required two separate groups to be conducted as staff were unable to travel between the two locations due to time and resource constraints. Participants provided written informed consent prior to taking part in the focus group. The focus groups were facilitated by AB.

The focus group topic guide was developed specifically for this study and refined by the research team and other experts, including researchers with qualitative research experience and training and clinical specialists in ED, midwifery and health visiting. The guide facilitated an informal discussion in each focus group on attitudes, knowledge, and clinical practice on identifying ED in pregnancy and in the postnatal period, focusing on the role of midwives and health visitors (see Additional file 2). Health professionals were not asked directly if they had a personal experience of ED.

### Participants

Thirty-three health professionals took part in the focus groups, the majority of whom were qualified health professionals (see Table 2). The sample was predominately white, female and over twenty-five years of age. The majority had trained in the UK and as part of that training had been educated in perinatal mental health, however only a small proportion had received training specifically in ED (*n* = 10; 29%).

**Table 2:** Study 2: Sample characteristics

Characteristics N (%)	*N* = 33
Age	
≤ 24	4 (12%)
25-35	11 (32%)
≧36	12 (35%)
*Missing*	*7*
Gender	
Female	32 (97%)
Male	1 (3%)
Ethnicity	
White	24 (73%)
Black	5 (15%)
Asian Indian	1 (3%)
Mixed ethnicity	1 (3%)
*Missing*	*2*
Professional category	
Student	
Midwife	5 (15%)
Health Visitor	5 (15%)
Qualified	
Midwife	14 (42%)
Health Visitor	9 (27%)
Training for current post in the UK	
Yes	28 (82%)
No	3 (9%)
*Missing*	*3*
Previous nurse training	
Yes	19 (56%)
No	12 (35%)
*Missing*	*2*
Received training in perinatal mental health	
Yes	24 (71%)
No	5 (15%)
*Missing*	*5*
Received training specifically in ED	
Yes	10 (29%)
No	21 (62%)
*Missing*	*3*

### Analysis

The focus group discussions were recorded and transcribed verbatim with identifying material removed. Data were analysed following the same procedure as detailed for Study 1 using a thematic analysis approach [33] to refine the emerging themes. The data were independently coded by two trained researchers (AB and MKM) and a percentage agreement of 79% was achieved by the rating-pair, with discrepancies resolved in the same manner as detailed for Study 1.

## Results

### Study 1

Women reported experiencing some improvements in ED symptoms during pregnancy, however over half of the sample reported experiencing any ED symptoms during pregnancy (*n* = 64; 63%), most common was calorie restriction and binge eating. Only a quarter (*n* = 26; 26%) of the sample reported disclosing their ED to a health professional involved in their antenatal care, and of the seventy-two (71%) women who did not disclose, seventy-one (70%) explained their reasons for not doing so (see Table 1). The findings generated five themes on the barriers to disclosure of ED in pregnancy as perceived by women: stigma, lack of opportunity, preference for self-management, current ED symptomatology and illness awareness (see Additional file 3).

#### Stigma

Stigma of ED was an important theme for women’s non-disclosure to a health professional. Many women reported that they felt shameful and embarrassed and feared judgement. Some women described feeling judged by health professionals based on their physical appearance, as illustrated by one woman who stated: *“I was overweight according to my BMI. I didn’t think they would believe me to tell them I had an actual problem. I was patronised by more than one healthcare professional who tried to educate me on nutrition. I got the impression they thought I was just lazy and ate junk food all of the time when this wasn’t the case. I felt they were too judgemental to approach”* (W42)*.* A few women expressed concern that a disclosure would lead to unwanted referrals to social services and other services: *“I would have been to worried to discuss with my midwife etc. for fear of being reprimanded for it (i.e. referred to social services”* (W49).

#### Lack of opportunity

Several women expressed a lack of opportunity to disclose and discuss an ED with a health professional. It was felt there was limited and insufficient enquiry by health professionals as “*they didn’t ask and it wasn’t raised as a concern”* (W67)*.* One woman expressed difficulties in establishing a rapport with a midwife that may have facilitated a disclosure: *“I didn’t have the same midwife for long enough to speak to them, it was rather stressful and upsetting”* (W21).

#### Preference for self-management

Some women reported not disclosing their ED to a health professional as they did not need or want specialist care and preferred to self-manage their disorder: *“I don’t like to talk about it and think I can manage on my own”* (W26), and *“I just wanted to deal with it myself”* (W36). In some cases, this feeling appeared to relate to how long their ED had been undetected for: *“I don’t really like to talk about it I have had some sort of disordered eating for a very long time it is very much part of me and no one else’s business”* (W27).

#### Current ED symptomatology

For some women disclosure was dependent on their current mental health status and perceived need to disclose a history of an ED to a health professional. A few women who experienced ED prior to becoming pregnant did not think it necessary to raise this with a healthcare professional: “*I didn’t think it was relevant as I have been OK for a few years now”* (W23). Other women reported improvements in ED symptoms during pregnancy so similarly did not feel it relevant to disclose: “*It wasn’t affecting me during my pregnancy, it helped*” (W50) and “*I felt like I was a lot better when I fell pregnant”* (W30).

#### Illness awareness

For several women, disclosure of an ED was dependent on their awareness of ED and acknowledging that their symptoms were that of an ED. This was particularly notable in women with BED as retrospectively some considered that they had dismissed their binge eating behaviours as general over eating:

*“Binge eating doesn’t seem like that big of an issue and I’ve never seen it as an eating disorder before”* W4.

*“I have only really just recognised that I have an issue & at the time I was pregnant did not realise. I just thought I was a greedy person”* W34.

### Study 2

Four main themes emerged on the barriers to identification of ED in the perinatal period as perceived by health professionals: system constraints, recognition of role, personal attitudes, and stigma and taboo (see Additional file 4).

#### System constraints

System constraints and associated sub-themes were the dominant theme affecting the identification of ED among health professionals. All the professionals reported receiving minimal, if any training on ED as part of their pre or post-registration clinical education, as one participant described: *“I know what an eating disorder is but I’ve not come across it through my health visitor training”* (P7). Some health professionals felt knowledge had to be inferred from other taught topics as ED were not specifically addressed, as illustrated by one qualified midwife involved in midwifery education: *“it wouldn’t be a module…it would be linked into mental ill health or BMI”* (P22). Several qualified and student participants reported receiving training, but considered that this was a general introduction to ED which was not specific to women during or after pregnancy and did not clarify their clinical role in identifying or managing ED: “*I don’t know whether it was actually mentioned apart from refer to a dietician, there wasn’t really any practical advice of what we need to do”* (P17). Some student midwives felt that module and programme leads expected knowledge of ED to be gained from self-directed learning or ‘learning’ in clinical practice: “*I think university often relies on us learning this kind of thing in practice that obviously we’ve got so much learning in the three years”* (P4).

Across all groups, most participants felt that the media was their main source of ED knowledge, with personal and previous clinical experience and training less likely to be described as a source. Most participants expressed limited understanding of ED beyond food-restriction associated with AN and self-induced vomiting associated with BN, and were not aware of implications for maternal and infant health. Some reported a lack of awareness that ED was classed as a mental health disorder, as one qualified health visitor explained: *“I have a very limited knowledge about the, those terms as in Bulimia and Anorexia, I’ve heard the words being thrown round quite a lot…but what I know as well is that it’s kind of linked…to mental health issues”* (P30). Consequently, many health professionals lacked evidence-based knowledge on ED which impacted on their confidence in enquiring and identifying ED. One qualified midwife described: *“it’s really hard when you’ve, when people give you information but you don’t know anything about it or there’s nothing much you can do”* (P17), and likewise this feeling was expected to affect disclosure by women: *“It’s that kind of feeling that, like a bit awkward and stuff like you don’t really know what to say and then it’s not going to help the women open up and discuss anymore with you”* (P4).

Health professionals related their poor awareness of relevant policies, guidance, care management plans and referral pathways to their lack of relevant training on ED: *“If it’s in the trust policy and guidelines I haven’t found it yet because I haven’t sort of come across it or it hasn’t been emphasised in the training”* (P3). Several qualified and student professionals reported not routinely including ED when asking women about their history of mental health problems: *“I never mention those words, I don’t think I ever ask a question that you know”* (P11). However, several midwives that had asked women felt *“there’s no point in asking the question if you don’t know what to say next”* (P13) referring to the lack of awareness on care pathways.

Midwives reported that time constraints in antenatal clinics would be likely to impact on their ability to enquire effectively about ED, with opportunities to screen for physical and mental health risk often limited to the initial pregnancy ‘booking’ appointment: “*these really big questions you know which can’t just be rushed over”* (P15). Some health visitors reported they would ask about women’s mental health at a ‘new baby’ visit which was usually allocated more time than other routine clinic appointments, but there was less focus on this being the main opportunity as *“when you see them for the first visit…you know the chances are you won’t see that person again…I’ve got someone who comes to clinic…the health visitor probably never saw her again anyway, whereas now it would be much more appropriate for me to say to her”* (P31).

In all groups, poor sharing of information about a woman’s physical and mental health was reported to be problematic. Qualified and student midwives reported limited means of relaying sensitive information or raising concerns about a woman’s mental health between colleagues. A woman’s pregnancy and medical history was expected to be documented in the woman’s handheld maternity notes, with concerns about confidentiality if a woman’s history of ED was included. Several midwives used domestic violence as an example of the limitations of using women’s handheld notes: *“obviously because they are handheld notes we’re very careful of what we write in them”* (P3). Communication between services particularly within primary care, for instance between the family doctor (GP), midwives and health visitors, was described by some qualified health visitors as limited, with services increasingly fragmented across acute and primary healthcare sectors: *“there was one midwife in every Sure Start and we were all attached so we would always be able to liaise with that midwife and they would liaise with us…now it’s like five midwives, like different midwives’ every time and they, they don’t build up that kind of rapport”* (P26). Sure Start is a UK parenting support programme, with centres primarily across England with slightly different versions in Wales, Scotland and Northern Ireland, but funding cuts have led to many of these centres closing in recent years [34]. Clinicians felt there were few opportunities to be involved in shared care as part of a multidisciplinary team, resulting in limited access to mental health expertise within or between services, particularly within health visiting: *“if you had some supervision around those sorts of issues, any sorts of issue where you’re just feeling like you’re holding something but you haven’t necessarily got the skills”* (P26). Furthermore, a few health visitors described poor awareness about ED generally among health professionals and not isolated to health visiting: *“I do really think that if we had it everybody else in the community teams would need it too because there would be no point in just training us if it then stopped with us”* (P27).

#### Recognition of role

Many clinicians were in favour of enquiring about ED but several considered their confidence and competence to identify complex mental health problems was limited, and their role should be more advisory and supportive. This was illustrated by one student midwife who described: “*we tell people what not to eat but not how do you eat*” (P3). Some qualified midwives felt that the primary focus in antenatal care was physical wellbeing of the woman and the fetus rather than the woman’s mental health: *“We would be just making sure that the baby was growing adequately… and then leaving the woman well alone in a way just focusing on the wellbeing of the baby”* (P11). The focus on the infant after the birth was similarly expressed by some of the qualified health visitors. A few student midwives considered whether women’s perceptions of their clinical roles could support or hinder a discussion about ED as *“a midwife…usually it’s for normal pregnancies, normality, and also is a figure that only she’s for the women and babies and the doctors maybe they seem, or the mental health services don’t sound probably very nice…maybe it’s easier because they know that this, the midwife is gonna follow them through the pregnancy”* (P2), whereas *“the health visitors kind of some were viewed for the baby and kind of for the child’s sake not someone to support the mum”* (P4).

#### Personal attitudes

The majority felt that health professionals needed to be empathic and positive so that women felt comfortable to raise and discuss their mental health problems: *“there is no room for negativity in midwifery”* (P12). However, one health visitor did express that she would feel uncomfortable to enquire about ED with women who were overweight compared to women who were underweight. One midwife discussed the need to recognise the health professional as an individual: *“we make assumptions that we all will deliver that health promotion message when actually attitudes and beliefs are integral to who we are, influence how we ask the question”* (P22).

#### Stigma and taboo

Health professionals in all groups discussed the stigma of ED, with some referring to it as a ‘taboo’ subject for women and clinicians, with less experienced midwives and health visitors describing greater anxiety about asking women. As one student midwife said: *“it does feel kind of sometimes like it’s one of those taboo questions a bit like domestic violence…but you kind of like skirt over like ‘you haven’t ever had any eating disorders, have you? No right moving on”* (P4). One qualified midwife explained: *“one needs to be sensitive about these things, mental health issues equals social services take away baby”* (P15).

## Discussion

This is the first research to specifically explore the perspectives of women and health professionals on the barriers to identifying ED during and after pregnancy. Our main findings were that perceived stigma had a major impact on women’s disclosure of their illness, and health professionals had low confidence in identifying ED as they lacked evidence-based knowledge and training. The discussion will mainly focus on these outcomes as they were the most prominent, and have direct implications to support effective identification of ED during and after pregnancy.

Consistent with general and pregnancy ED research, women were often reluctant to disclose their ED to a health professional [25, 29]. Stigma was a key barrier reported by the women in this research, and health professionals similarly felt it hindered discussion and enquiry about ED. Stigma is consistently implicated as a barrier to disclosure and treatment-seeking in the ED and wider mental health literature [27, 28, 35]. The term stigma involves perceived and experienced stereotyping, prejudice and discrimination to the detriment of the targeted group [36]. The stigma of mental health is widely recognised, but it can be greater for ED as sufferers are perceived as more responsible and in control of their ED behaviours [37, 38]. The stigmatising attitudes towards BED specifically are comparable to attitudes towards obesity and overweight status [39]. Weight stigma is well recognised and reinforced by some of the more pervasive anti-obesity campaigns [39]. Given the association between BED and obesity [40], the stigma women with BED experience may be compounded by their weight status. Stigmatising attitudes can contribute to feelings of shame and guilt, which cause an individual to feel personal responsibility for their behaviours. As a consequence, an individual may want to hide their disorder [41], avoid disclosure and show reluctance to seek help for their ED [27–29].

In the UK, several campaigns have been launched to raise public awareness and reduce stigma about mental health, such as the ‘Time to Change’ campaign [42], and several initiatives have been launched to address perinatal mental health awareness specifically, such as the ‘Better Births’ initiative [43]. Similar anti-stigma programmes exist in other countries, such as ‘Beyond Blue’ in Australia [44], ‘Mental Health Commission of Canada’ [45], and ‘Bring Change 2 Mind’ in the US [46], and these, along with other campaigns, have formed a global alliance to reduce mental health stigma [47]. However, ED continue to be largely neglected in these mental health awareness campaigns. BED is also not considered and neither is the broader issue of weight stigma within the anti-obesity and healthy eating campaigns and clinical guidance [48–50]. The campaigns and clinical guidance [48–50] aimed at addressing the rising prevalence of obesity focus on changing nutrition but not on eating disorder behaviours that might affect nutrition. Considering the high level of comorbidity [40], the identification of ED perinatally may subsequently support obesity prevention in pregnancy and postnatally. Initiatives are needed to specifically target and address the broad range of ED to reduce stigma, prevent discrimination and raise awareness. Only by raising the profile of ED and reducing stigma will disclosure and open discussion with health professionals be encouraged among women during and after pregnancy who suffer these mental health disorders.

Health professionals considered an important barrier to identifying ED was their lack of evidence-based knowledge and training, which subsequently impacted on their confidence. This finding was not unexpected as ED are not currently specified in the core clinical competencies required as part of pre-registration training of midwives and health visitors in the UK [51, 52]. This reflects poor integration of available guidance in to pre and post-registration training [24]. Future curriculum revisions need to educate about the complexity and range of ED and ED behaviours, and include the changes in symptoms that may be experienced during and after pregnancy [3, 6–9]. Research is needed to explore facilitators to disclosure to identify acceptable means of enquiry and management that are sensitive and responsive to the needs of women, and findings need to be incorporated in to future training. Training also needs to address individual attitudes about ED given the expectation for health professionals to deliver care in a consistent manner.

Other system level barriers to identifying ED in pregnancy and postnatally were identified, specifically poor continuity of care and poor communication between health professionals and women, and between health professionals. Women and health professionals described the lack of opportunity and time within routine antenatal and postnatal care contacts to discuss ED in a comfortable way to encourage disclosure, with midwives advocating the potential benefit of a ‘case loading’ model of midwifery care. This model of midwifery care is considered to promote better continuity of care in pregnancy and is advocated in UK policy and guidance [53, 54] and other countries with similar healthcare systems such as Australia [55]. Case loading models of midwifery care have been associated with better maternal and infant outcomes, patient satisfaction, and cost effectiveness in comparison to other models of maternity care [56–58], yet provision remains variable [59, 60]. Poor communication between health professionals was similarly an important barrier as methods of relaying concern about women were complicated by the need to balance communication of crucial information with respecting the sensitive and confidential nature of disclosure. The issue of communication of risk between acute and primary care services is increasingly affected by fragmented and poorly integrated maternity services, particularly in primary care settings with services central to coordinating healthcare [61]. Addressing these system level barriers could promote an environment conducive to open discussion and support the role of the health professionals in the identification of ED in the perinatal period.

### Strengths and limitations

Strengths of this research include the exploration of experiences from both women and health professionals, with consistency in the findings between the two groups. Research findings are likely to be relevant for women who have ED regardless of place of birth, with issues for health professional training likely to be relevant in settings where women can access care from midwives and health visitors or an equivalent health professional.

There are several limitations to the research to be taken in to account when considering the implications of the findings. Convenience sampling was necessary for logistical reasons however, it limits the representativeness of the sample and generalisability of the findings. There may have been recall bias as most women were reflecting on past experiences of antenatal care. The single eligibility question may have been ambiguous in the absence of a clinical diagnosis and did not distinguish between past or current ED. However this type of self-report indicator was used primarily for practical reasons, further it has been validated in an antenatal sample [62] and no ED screening measures have been validated in pregnancy. Future research could focus on women who have been clinically diagnosed with ED.

## Conclusions

There are several important barriers to the identification of ED in pregnancy and the postnatal period. Stigma had a major impact on women’s disclosure of their illness, whilst health professionals had poor confidence in identifying ED as they lacked evidence-based knowledge and training. The need to identify perinatal mental health problems has been increasingly recognised, however ED continue to be neglected and it is important to raise awareness with health professionals. Similarly, clinical guidance aimed at responding to the rising prevalence of obesity focus on changing nutrition but not on ED behaviours that might affect nutrition. Improving ED education and training for health professionals may contribute to reducing stigma and increase confidence in identifying ED. The barriers identified in this research need to be addressed if recognition and response to women with ED during the perinatal period is to improve.

## Additional file

Additional file 1: Study 1: Mixed-measures survey. (DOCX 13 kb)

Additional file 2: Study 2: Focus group topic guide. (DOCX 16 kb)

Additional file 3: Study 1: Themes with illustrative quotations. (DOCX 14 kb)

Additional file 4: Study 2: Themes with illustrative quotations. (DOCX 15 kb)

## Abbreviations

AN: Anorexia Nervosa
BED: Binge Eating Disorder
BN: Bulimia Nervosa
ED: Eating Disorders
NHS: National Health Service
NICE: National Institute for Health and Care Excellence
