# Supplementary material for: Barriers to identifying eating disorders in pregnancy and in the postnatal period: a qualitative approach
Source: BMC Pregnancy Childbirth. 2018 May 15;18:114. doi: 10.1186/s12884-018-1745-x (PMC5952825; doi:10.1186/s12884-018-1745-x)
Supplement: Supplementary file 1 — Study 1: Mixed-measures survey. (DOCX 13 kb) [file 12884_2018_1745_MOESM1_ESM.docx]

| **Additional file 1. Study 1: Mixed-measures survey** |  |
| --- | --- |
| 1. Do you have or have you had an eating disorder? | Yes |
|  | No |
| 1. Which eating disorder did you have during or around the time of your pregnancy? | Anorexia nervosa |
|  | Bulimia nervosa |
|  | Binge eating disorder |
|  | Eating disorder not otherwise specified (EDNOS) |
| 1. If any, which eating disorder symptoms did you experience during pregnancy? | No. I wasn’t unwell at the time |
|  | Purging |
|  | Binging |
|  | Calorie or food restriction |
|  | Exercising |
|  | Low weight |
| 1. Did any of these eating disorder symptoms improve during pregnancy? | Purging |
|  | Binging |
|  | Calorie/food restriction |
|  | Exercising |
|  | Low weight |
| 1. Were the health professionals you saw during your pregnancy aware of your eating disorder? | Yes |
|  | No |
|  | Unsure |
| 1. Did you let the health professionals know or talk to them about having an eating disorder? | Yes |
|  | No |
|  |  |
| 1. If you did not talk to the health professionals, what was the reason(s)? | *Open-ended response* |
|  |  |
