# Supplementary material for: Barriers to identifying eating disorders in pregnancy and in the postnatal period: a qualitative approach
Source: BMC Pregnancy Childbirth. 2018 May 15;18:114. doi: 10.1186/s12884-018-1745-x (PMC5952825; doi:10.1186/s12884-018-1745-x)
Supplement: Supplementary file 2 — Study 2: Focus group topic guide. (DOCX 16 kb) [file 12884_2018_1745_MOESM2_ESM.docx]

**Additional file 2. Study 2: Focus group topic guide**

1. From your training, what do you know about eating disorders?
2. From your training, what do you know about the impact of eating disorders on pregnancy and birth outcomes, including the postnatal period?
3. From your training, what specialised support do you think pregnant women and mothers with eating disorders may need?
4. Based on your experience, what do you think about the current training on eating disorders?
5. Based on your experience, what do you think about routine screening for eating disorders in pregnant and postnatal women?
6. Based on your experience, do you think midwives and health visitors should be involved in the care management plans for pregnant and postnatal women with eating disorders?
