# Supplementary material for: Barriers to identifying eating disorders in pregnancy and in the postnatal period: a qualitative approach
Source: BMC Pregnancy Childbirth. 2018 May 15;18:114. doi: 10.1186/s12884-018-1745-x (PMC5952825; doi:10.1186/s12884-018-1745-x)
Supplement: Supplementary file 3 — Study 1: Themes with illustrative quotations. (DOCX 14 kb) [file 12884_2018_1745_MOESM3_ESM.docx]

**Additional file 3. Study 1: Themes with illustrative quotations**

| **Themes** | **Subthemes** | **Illustrative quotations** |
| --- | --- | --- |
| Stigma | Feel shame and embarrassment | *“Embarrassed and ashamed”* W13  *“I was overweight according to my BMI. I didn't think they would believe me to tell them I had an actual problem. I was patronised by more than one healthcare professional who tried to educate me on nutrition. I got the impression they thought I was just lazy and ate junk food all of the time when this wasn't the case. I felt they were too judgemental to approach”* W42  *“Shame, I didn't want to be told off or for them to think I was a bad mum”* W64  *“Shame. I wasn't skinny enough to back it up. I looked normal and fat”* W65 |
|  | Fear of adverse consequences | *“Fear of intervention”* W17  *“I would have been to worried to discuss with my midwife etc. for fear of being reprimanded for it (i.e. referred to social services”* W49 |
| Lack of opportunity | Limited and insufficient enquiry | *“I expected them to know and bring it up but they never did”* W28    *“they didn't ask and it wasn't raised as a concern”* W67  *“Was never mentioned or discussed”* W70 |
|  | Establishing rapport with the midwife | *‘I didn't have the same midwife for long enough to speak to them, it was rather stressful and upsetting’* W21 |
| Preference for self-management |  | *“I don't like to talk about it and think I can manage on my own”* W26  *“I don’t really like to talk about it I have had some sort of disordered eating for a very long time it is very much part of me and no one else’s business”* W27    *“I just wanted to deal with it myself”* W36  *“I've never had a diagnosis so why start now”* W37 |
| Current symptomatology |  | *“I didn't think it was relevant as i have been OK for a few years now”* W23  *“I thought I was past the worst of it”* W38    *“I wasn't unwell at the time so didn't seem like something which needed bringing up as they didn't ask”* W44  *“It wasn't affecting me during my pregnancy, it helped”* W50 |
| Illness awareness |  | *“Binge eating doesn't seem like that big of an issue and I've never seen it as an eating disorder before”* W4  *“I have only really just recognised that I have an issue & at the time I was pregnant did not realise. I just thought I was a greedy person”* W34 |
