# Supplementary material for: Barriers to identifying eating disorders in pregnancy and in the postnatal period: a qualitative approach
Source: BMC Pregnancy Childbirth. 2018 May 15;18:114. doi: 10.1186/s12884-018-1745-x (PMC5952825; doi:10.1186/s12884-018-1745-x)
Supplement: Supplementary file 4 — Study 2: Themes with illustrative quotations. (DOCX 15 kb) [file 12884_2018_1745_MOESM4_ESM.docx]

**Additional file 4. Study 2: Themes with illustrative quotations**

| **Themes** | **Subthemes** | **Illustrative quotations** |
| --- | --- | --- |
| System constraints | Lack of evidence-based knowledge | *“we got lecturers about healthy eating and what we recommend applies to women, what they can eat but nothing, nothing related to eating disorders, it was obesity”* P2    *“I think university often relies on us learning this kind of thing in practice that obviously we’ve got so much learning in the 3 years”* P4  *“It's that kind of feeling that, like a bit awkward and stuff like you don’t really know what to say and then it’s not going to help the women open up and discuss anymore with you”* P4  *“I know what an eating disorder is but I’ve not come across it through my health visitor training”* P7  *“I don’t know whether it was actually mentioned apart from refer to a dietician, there wasn’t really any practical advice of what we need to do” P17*  *“it’s really hard when you’ve, when people give you information but you don’t know anything about it or there’s nothing much you can do”* P17  *“it wouldn’t be a module…it would be linked into mental ill health or BMI”* P22  *“I have a very limited knowledge about the, those terms as in Bulimia and Anorexia, I’ve heard the words being thrown round quite a lot…but what I know as well is that it’s kind of linked…to mental health issues”* P30 |
|  | Lack of policies, guidance and care pathways | *“I don’t think even in the box where you select if they say yes they have, it even has eating disorders”* P1  *“If it’s in the trust policy and guidelines I haven’t found it yet because I haven’t sort of come across it or it hasn’t been emphasised in the training”* P3  *“I never mention those words, I don’t think I ever ask a question that you know”* P11  *“there’s no point in asking the question if you don’t know what to say next”* P13 |
|  | Time constraints | *“these really big questions you know which can’t just be rushed over”* P15  *“when you see them for the first visit…you know the chances are you won’t see that person again…I’ve got someone who comes to clinic…the health visitor probably never saw her again anyway, whereas now it would be much more appropriate for me to say to her”* P31 |
|  | Communication within and between services | *“obviously because they are handheld notes we’re very careful of what we write in them”* P3  *“there was one midwife in every Sure Start and we were all attached so we would always be able to liaise with that midwife and they would liaise with us…now it’s like five midwives, like different midwives’ every time and they, they don’t build up that kind of rapport”* P26  *“I’ve had quite a few in only the last few months of being qualified where there’s things that we should of known about and didn’t”* P27  *“I didn’t know her history ‘cos she just moved into the area”* P33 |
|  | Lack of support | *“I mean having tried to refer to safeguarding for an eating problem before, it came straight back to me and they weren’t interested at all”* P27  *“if you had some supervision around those sorts of issues, any sorts of issue where you’re just feeling like you’re holding something but you haven’t necessarily got the skills”* P26  *“I do really think that if we had it everybody else in the community teams would need it too because there would be no point in just training us if it then stopped with us”* P27 |
| Recognition of role | Role in mental health screening | *“a midwife…usually it’s for normal pregnancies, normality, and also is a figure that only she’s for the women and babies and the doctors maybe they seem, or the mental health services don’t sound probably very nice…maybe it’s easier because they know that this, the midwife is gonna follow them through the pregnancy”* P2  *“we tell people what not to eat but not how do you eat”* P3  *“the health visitors kind of some were viewed for the baby and kind of for the child’s sake not someone to support the mum”* P4  *“yes a sensitive issue obviously but you know, we’re in that prime position aren’t we, we’re able to so that”* P14 |
|  | Focus on physical wellbeing | *“We would be just making sure that the baby was growing adequately… and then leaving the woman well alone in a way just focusing on the wellbeing of the baby”* P11 |
| Personal attitudes | Empathy | *“there is no room for negativity in midwifery”* P12 |
|  | Personal attitude | *“we make assumptions that we all will deliver that health promotion message when actually attitudes and beliefs are integral to who we are, influence how we ask the question”* P22 |
| Stigma and taboo |  | *“it does feel kind of sometimes like it’s one of those taboo questions a bit like domestic violence…but you kind of like skirt over like ‘you haven’t ever had any eating disorders, have you? No right moving on”* P4  *“it’s quite a big kind of word isn’t it for women, no one wants to have a mental health problem do they, a bit of a stigma”* P12  *“one needs to be sensitive about these things, mental health issues equals social services take away baby”* P15 |
